# The clinical impact of concomitant medication use on the outcome of postoperative recurrent non-small-cell lung cancer in patients receiving immune checkpoint inhibitors

**DOI:** 10.1371/journal.pone.0263247

**Published:** 2022-02-07

**Authors:** Kazuki Takada, Mototsugu Shimokawa, Shinkichi Takamori, Shinichiro Shimamatsu, Fumihiko Hirai, Yuki Ono, Tetsuzo Tagawa, Tatsuro Okamoto, Motoharu Hamatake, Isamu Okamoto, Masaki Mori

**Affiliations:** 1 Department of Thoracic Surgery, Kitakyushu Municipal Medical Center, Kokurakita-ku, Kitakyushu, Fukuoka, Japan; 2 Department of Biostatistics, Yamaguchi University Graduate School of Medicine, Ube, Yamaguchi, Japan; 3 Clinical Research Institute, National Hospital Organization Kyushu Cancer Center, Minami-ku, Fukuoka, Fukuoka, Japan; 4 Department of Thoracic Oncology, National Hospital Organization Kyushu Cancer Center, Minami-ku, Fukuoka, Fukuoka, Japan; 5 Department of Surgery and Science, Graduate School of Medical Sciences, Kyushu University, Higashi-ku, Fukuoka, Fukuoka, Japan; 6 Research Institute for Diseases of the Chest, Graduate School of Medical Sciences, Kyushu University, Higashi-ku, Fukuoka, Fukuoka, Japan; Chung Shan Medical University, TAIWAN

## Abstract

A recent study suggested that proton pump inhibitor (PPI) use in patients with advanced non-small-cell lung cancer (NSCLC) receiving immune checkpoint inhibitors (ICIs) was associated with poor clinical outcomes. However, the clinical impact of PPI use on the outcome of patients receiving ICIs for postoperative recurrent NSCLC is unknown. The outcomes of 95 patients with postoperative recurrence of NSCLC receiving ICIs at 3 medical centers in Japan were analyzed. We conducted adjusted Kaplan–Meier survival analyses with the log-rank test, a Cox proportional hazards regression analysis, and a logistic regression analysis using inverse probability of treatment weighting (IPTW) to minimize the bias arising from the patients’ backgrounds. The IPTW-adjusted Kaplan–Meier curves revealed that the progression-free survival (PFS), but not the overall survival (OS), was significantly longer in patients who did not receive PPIs than in those who did receive them. The IPTW-adjusted Cox regression analysis revealed that PPI use was an independent poor prognostic factor for the PFS and OS. Furthermore, in the IPTW-adjusted logistic regression analysis, PPI non-use was an independent predictor of disease control. In this multicenter and retrospective study, PPI use was associated with poor clinical outcomes in patients with postoperative recurrence of NSCLC who were receiving ICIs. PPIs should not be prescribed indiscriminately to patients with postoperative recurrence of NSCLC who intend to receive ICIs. These findings should be validated in a future prospective study.

## Introduction

Immune checkpoint inhibitors (ICIs) have been approved for use in patients with advanced or recurrent non-small-cell lung cancer (NSCLC), and they are the standard treatment options for these patients globally. Programmed cell death-ligand 1 (PD-L1) tumor expression is a strong predictor for selecting patients who might benefit from ICIs. However, the identification of reliable biomarkers other than PD-L1 tumor expression is considered crucial, and many potential predictive markers for ICI efficacy in NSCLC have been examined.

Recently, several studies have revealed the influence of the gastrointestinal microbiota on the response to cancer immunotherapy [1–3]. Therefore, drugs associated with gastrointestinal dysbiosis and bacterial richness, such as antibiotics, proton pump inhibitors (PPIs), and probiotics, might affect the efficacy of ICIs [1–5]. Antibiotics are representative drugs that affect the gastrointestinal microbiome, which may be an important factor associated with the immune response [1–3]. PPIs also affect the gastrointestinal microbiome, and several studies have shown that the changes induced by PPIs were more prominent than those induced by antibiotics [4–6]. PPI use is associated with gastrointestinal dysbiosis, decreased bacterial richness, and promotion of T cell tolerance [4, 5]. Therefore, PPI use may be associated with the efficacy of ICIs, and these drugs may reduce the efficacy of ICIs. According to previous reports, the use of antibiotics and PPIs were significantly associated with poor clinical outcomes in patients with advanced-stage cancers, including NSCLC, who received ICIs [1–3, 7–9]. However, the clinical impact of these medications on the outcomes of postoperative recurrent NSCLC in patients receiving ICIs is unknown. The survival outcome differs between advanced-stage cancers and postoperative recurrent cancers in NSCLC [10, 11]. Therefore, it is important to independently conduct analyses in patients with advanced-stage cancers and those with postoperative recurrent cancers in NSCLC. The present findings might be informative for clinicians, including thoracic surgeons, involved in the treatment of patients with NSCLC.

We investigated the influence of these medications, especially PPIs, on the clinical outcomes of postoperative recurrent NSCLC in patients receiving ICIs in this study.

## Materials and methods

### Patients and samples in this study

All procedures performed in this study involving human participants were in accordance with the ethical standards of the institutional and/or national research committee and with the 1964 Declaration of Helsinki and its later amendments or comparable ethical standards. The current study was a retrospective cross-sectional one and was approved by our institutional review boards (Kyushu University, IRB No. 2020–76, Kyushu Cancer Center, IRB No. 2019–45, and Kitakyushu Municipal Medical Center, IRB No. 202008008). The requirement for informed consent from the patients enrolled in this study was waived because of the retrospective design of the study, and patient information was protected.

We retrospectively identified and enrolled 95 patients with postoperative recurrent NSCLC receiving ICIs (nivolumab, pembrolizumab, or atezolizumab monotherapy or combination therapy) between January 2016 and December 2019 at 3 medical centers in Japan: Kyushu University Hospital, National Hospital Organization Kyushu Cancer Center, and Kitakyushu Municipal Medical Center. We examined the following patients’ clinicopathological features in this study: age at the time of treatment initiation, sex, Eastern Cooperative Oncology Group (ECOG) performance status (PS), smoking history, driver oncogene mutation status, histology, PD-L1 expression status, and medications (probiotics and PPIs). We were unable to obtain sufficient information about antibiotic use from patients’ medical records, and we did not include the factor in this study. Probiotics included *Bifidobacterium*, *Clostridium butyricum*, and antibiotic-resistant lactic acid bacteria, and PPIs included omeprazole, lansoprazole, rabeprazole, esomeprazole, and vonoprazan fumarate. For both probiotics and PPIs, their use at the time of treatment initiation was examined, and whether or not these medications continued to be prescribed afterward was not investigated in this study. We usually assessed the tumor response by computed tomography every six to eight weeks according to the Response Evaluation Criteria in Solid Tumors (RECIST), version 1.1 [12], defining “complete response (CR) + partial response (PR) + stable disease (SD)” as "disease control" according to the RECIST criteria. We obtained all clinical information and follow-up data from patients’ medical records, and the end of the follow-up period of this study was April 30, 2020.

### PD-L1, epidermal growth factor receptor (EGFR), and anaplastic lymphoma kinase (ALK) analyses

PD-L1 immunohistochemistry was performed using the pharmDx antibody (clone 22C3, Dako North America, Inc., Agilent/Dako, Carpinteria, CA, USA) [13]. PD-L1 data was categorized using the tumor proportion score (TPS). The *EGFR* status was assessed in tumor samples using the peptide nucleic acid-locked nucleic acid polymerase chain reaction clamp method (Mitsubishi Chemical Medience, Tokyo, Japan) [14], and the *ALK* status was determined in tumor tissue using fluorescence *in situ* hybridization (FISH) with a Vysis ALK Break Apart FISH Probe Kit (Abbott Molecular, Des Plaines, IL, USA) [15]. We extracted all data on the PD-L1 tumor expression and mutation status of driver oncogenes (*EGFR* and *ALK*) from patients’ medical records.

### Statistical analyses

All statistical analyses in this study were performed using the JMP^®^ 14.0 or SAS^®^ 9.4 (SAS Institute, Cary, NC, USA) software programs, and *P* < 0.05 was considered statistically significant.

Patient demographics and baseline characteristics were summarized using descriptive statistics or contingency tables. We conducted adjusted Kaplan–Meier survival analyses with the log-rank test, a Cox proportional hazards regression analysis, and a logistic regression analysis using inverse probability of treatment weighting (IPTW) to minimize the bias arising from the patients’ backgrounds in this study [16, 17]. Associations between PPI use and clinical factors before and after weighting were examined using the χ^2^ test. We defined the progression-free survival (PFS) and overall survival (OS) as follows: the PFS was defined as the period from the initial treatment to clinical or radiographic progression or death, and the OS was defined as the period from the initial treatment to the date of last follow-up or death. We constructed survival curves using the Kaplan–Meier method with the log-rank test. A Cox proportional hazards regression analysis was used to estimate the hazard ratios for risk factors. Univariate and multivariate analyses of the relationships between disease control and clinical factors were performed via a logistic regression analysis. The backward elimination method was used in the multivariate analyses of the PFS, OS, and relationship between disease control and clinical factors. The model was run with all variables, and the variable with the highest *P* value was excluded. This process was repeated until all remaining variables yielded *P* values of < 0.05.

## Results

### Patient characteristics in this study

**Fig 1:**
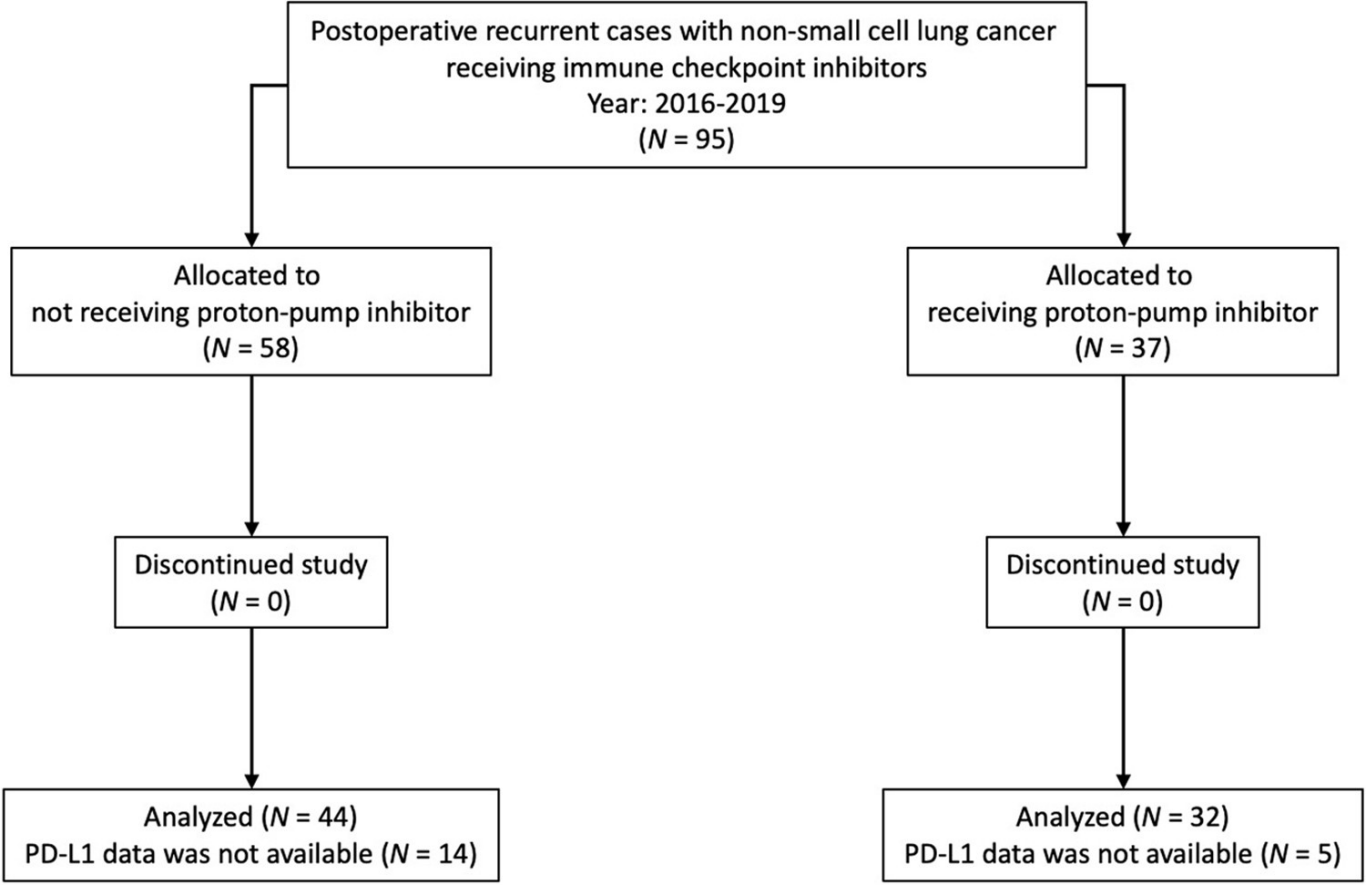
CONSORT diagram. CONSORT diagram in this study.

**Table 1** shows the clinical characteristics of the 95 patients included in this study. Among the 95 patients, 12 (12.6%) and 37 (38.9%) received probiotics and PPIs, respectively. *EGFR* or *ALK* status data were available for 82 patients (86.3%), and PD-L1 tumor expression data were available for 76 patients (80.0%). The CONSORT diagram in this study is presented in **Fig 1**, and the characteristics of the patients before and after weighting are shown in **Table 2**. Important factors associated with the efficacy of ICIs, namely the smoking history, driver oncogene mutation status, and PD-L1 tumor expression status, were used in the IPTW treatment-allocation model. The standardized mean differences of the whole model before and after weighting were 0.4257 and −0.0527, respectively.

**Table 1:** Clinicopathological characteristics of all patients (*N* = 95).

Factors		Value or no. of patients
Age (years)	Median	69
	Maximum and minimum	88, 43
Sex	Female	17 (17.9%)
	Male	78 (82.1%)
ECOG PS	0	53 (55.8%)
	1	36 (37.9%)
	2	4 (4.2%)
	3	2 (2.1%)
Smoking history	Never-smoker	17 (17.9%)
	Ex-smoker	61 (64.2%)
	Current smoker	17 (17.9%)
Mutation status (*EGFR* or *ALK*)	Wild-type	72 (75.8%)
	Mutant^a^	10 (10.5%)
	Unknown	13 (13.7%)
Histology	Adenocarcinoma	66 (69.5%)
	Squamous cell carcinoma	20 (21.0%)
	Others or unknown^b^	9 (9.5%)
Immune checkpoint inhibitor	Nivolumab	41 (43.2%)
	Pembrolizumab	34 (35.8%)
	Pembrolizumab + chemotherapy	10 (10.5%)
	Atezolizumab	9 (9.5%)
	Atezolizumab + chemotherapy	1 (1.0%)
PD-L1 tumor proportion score	<1%	23 (24.2%)
	≥1% and <50%	24 (25.3%)
	≥50%	29 (30.5%)
	Unknown	19 (20.0%)
Probiotics	No	83 (87.4%)
	Yes	12 (12.6%)
Proton pump inhibitor	No	58 (61.1%)
	Yes	37 (38.9%)

^a^Ten patients were *EGFR*-positive.

^b^Eight patients with sarcomatoid carcinoma and one patient with adenosquamous carcinoma.

*ALK*, anaplastic lymphoma kinase; ECOG, Eastern Cooperative Oncology Group; *EGFR*, epidermal growth factor receptor; PD-L1, programmed cell death-ligand 1; PS, performance status.

**Table 2:** Characteristics of the patients according to the use of PPIs before and after weighting.

Factors		Unweighted, *N* (%)				Weighted, %			
	PPI	No	Yes	*P* value	SMD	No	Yes	*P* value	SMD
Age (years)	<65	15 (34.1%)	9 (28.1%)	0.5807	−0.1274	34.1%	24.9%	0.3444	−0.1958
	≥65	29 (65.9%)	23 (71.9%)			65.9%	75.1%		
Sex	Female	9 (20.5%)	4 (12.5%)	0.3632	0.2128	20.5%	26.0%	0.5374	−0.1482
	Male	35 (79.5%)	28 (87.5%)			79.5%	74.0%		
ECOG PS	0	26 (59.1%)	18 (56.3%)	0.8044	−0.0567	59.1%	49.6%	0.3696	−0.1898
	1–3	18 (40.9%)	14 (43.7%)			40.9%	50.4%		
Smoking history	Never-smoker	10 (22.7%)	3 (9.4%)	0.1270	0.3699	22.7%	23.8%	0.9027	−0.0305
	Smoker	34 (77.3%)	29 (90.6%)			77.3%	76.2%		
Mutation status (*EGFR* or *ALK*)	Others	10 (22.7%)	5 (15.6%)	0.4425	−0.1811	22.7%	25.6%	0.7562	0.0721
	Wild-type	34 (77.3%)	27 (84.4%)			77.3%	74.4%		
Histology	Non-Sq	36 (81.8%)	26 (81.3%)	0.9497	−0.0144	81.8%	82.6%	0.9249	0.0195
	Sq	8 (18.2%)	6 (18.7%)			18.2%	17.4%		
PD-L1 tumor proportion score	<50%	26 (59.1%)	21 (65.6%)	0.5626	−0.1352	59.1%	59.3%	0.9821	−0.0049
	≥50%	18 (40.9%)	11 (34.4%)			40.9%	40.7%		
Probiotics	No	41 (93.2%)	27 (84.4%)	0.2168	−0.2777	93.2%	81.0%	0.0882	−0.3837
	Yes	3 (6.8%)	5 (15.6%)			6.8%	19.0%		

*ALK*, anaplastic lymphoma kinase; ECOG, Eastern Cooperative Oncology Group; *EGFR*, epidermal growth factor receptor; PD-L1, programmed cell death-ligand 1; PPI, proton pump inhibitor; PS, performance status; SMD, standardized mean difference; Sq, squamous cell carcinoma.

**S1 Table** shows the breakdown ratios of the PPIs used for patients in this study, and **S2 Table** summarizes the reasons for PPI use in the 37 patients. Approximately half of these patients (*N* = 18 [48.7%]) were prescribed PPIs without obvious justification.

### Effects of PPI use on the survival

First, we investigated the effects of PPI use on the survival using the original values. The median follow-up time was 331 days (range, 7–1363). The Kaplan–Meier curves revealed that patients who did not receive PPIs had a significantly longer PFS, but not OS, than those treated with PPIs (*P* = 0.0163 and *P* = 0.0555, respectively; **S1A** and **S1B Fig**). Multivariate analyses revealed that the ECOG PS, mutation status, PPI use, and PD-L1 expression status were independent prognostic factors for the PFS (*P* = 0.0075, *P* = 0.0316, *P* = 0.0002, and *P* = 0.0047, respectively; **S3 Table**), whereas the ECOG PS and histology were independent prognostic factors for the OS (*P* = 0.0018 and *P* = 0.0042, respectively; **S3 Table**).

**Fig 2:**
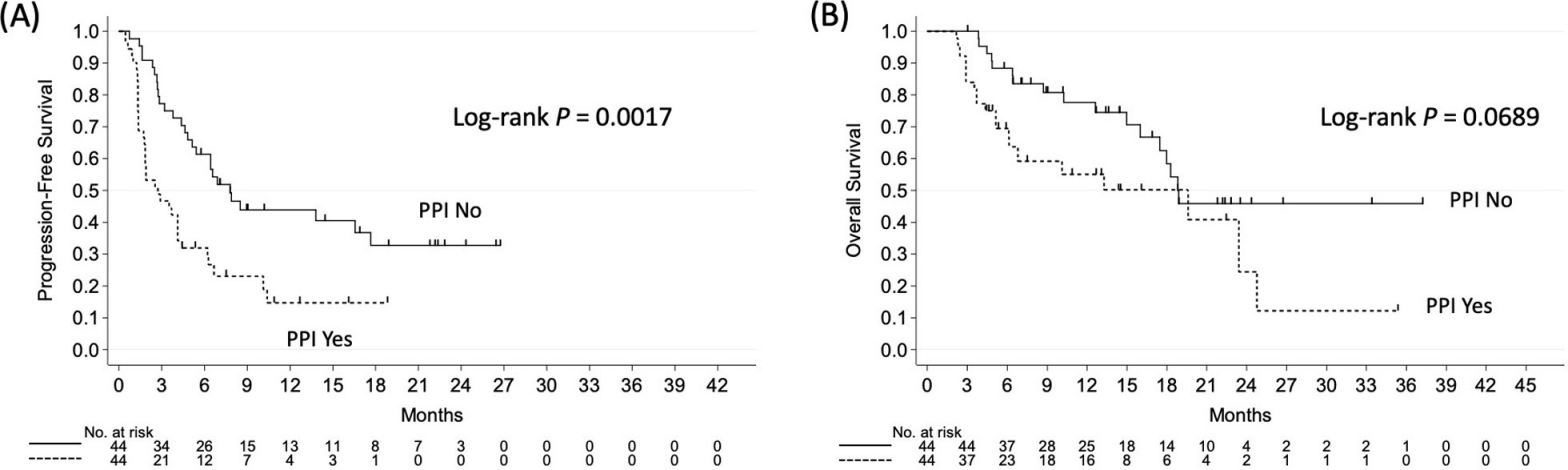
The inverse probability of treatment weighting-adjusted Kaplan–Meier curves according to the use or non-use of proton pump inhibitors. (A) The progression-free survival and (B) the overall survival.

Next, we investigated the effects of PPI use on the survival using IPTW-adjusted values. The IPTW-adjusted Kaplan–Meier curves showed that patients who did not receive PPIs had a significantly longer PFS, but not OS, than those treated with PPIs (*P* = 0.0017 and *P* = 0.0689, respectively; **Fig 2A** and **2B**). The IPTW-adjusted Cox analyses showed that the ECOG PS, mutation status, PPI use, and PD-L1 expression status were independent prognostic factors for the PFS (*P* = 0.0021, *P* = 0.0006, *P* < 0.0001, and *P* = 0.0009, respectively; **Table 3**), whereas the ECOG PS, mutation status, probiotics use, and PPI use were independent prognostic factors for the OS (*P* = 0.0499, *P* = 0.0202, *P* = 0.0076, and *P* = 0.0061, respectively; **Table 3**). As shown in **Table 3**, PPI use had a stronger impact on the survival than the PD-L1 expression status.

**Table 3:** Inverse probability of treatment weighting-adjusted univariate and multivariate analyses of PFS and OS.

Factors		PFS					OS			
		Univariate analysis		Multivariate analysis		Univariate analysis			Multivariate analysis	
		HR (95% CI)	*P* value	HR (95% CI)	*P* value	HR (95% CI)		*P* value	HR (95% CI)	*P* value
Age (years)	≥65/<65	1.12 (0.65–1.94)	0.6820			1.04 (0.53–2.04)		0.9167		
Sex	Female/Male	1.80 (1.04–3.12)	0.0350			0.85 (0.37–1.97)		0.7079		
ECOG PS	1–3/0	2.41 (1.46–4.00)	0.0006	2.25 (1.34–3.78)	0.0021	1.44 (0.77–2.70)		0.2598	1.94 (1.00–3.75)	0.0499
Smoking history	Never-smoker/Smoker	1.68 (0.96–2.92)	0.0673			0.63 (0.26–1.54)		0.3118		
Mutation status (*EGFR* or *ALK*)	Others/Wild-type	2.25 (1.30–3.91)	0.0039	2.96 (1.60–5.47)	0.0006	1.79 (0.88–3.66)		0.1079	2.46 (1.15–5.25)	0.0202
Histology	Sq/Non-Sq	1.55 (0.85–2.83)	0.1520			2.78 (1.43–5.39)		0.0026		
Probiotics	No/Yes	1.15 (0.52–2.52)	0.7279			2.75 (0.83–9.14)		0.0984	5.93 (1.60–21.92)	0.0076
Proton pump inhibitor	Yes/No	2.34 (1.41–3.90)	0.0011	4.12 (2.28–7.46)	<0.0001	1.94 (1.02–3.67)		0.0425	2.55 (1.31–4.99)	0.0061
PD-L1 tumor proportion score	<50%/≥50%	2.52 (1.47–4.31)	0.0008	2.64 (1.49–4.68)	0.0009	1.50 (0.77–2.95)		0.2376		

*ALK*, anaplastic lymphoma kinase; CI, confidence interval; ECOG, Eastern Cooperative Oncology Group; *EGFR*, epidermal growth factor receptor; HR, hazard ratio; OS, overall survival; PD-L1, programmed cell death-ligand 1; PFS, progression-free survival; PS, performance status; Sq, squamous cell carcinoma.

### Effects of PPI use on disease control

Finally, we examined the associations between disease control and clinical factors. The disease control status was CR, PR, SD, and disease progression in 2 (2.1%), 21 (22.1%), 26 (27.4%), and 40 patients (42.1%), respectively, and the status was not evaluable in 6 patients (6.3%). Therefore, the objective response (CR + PR) and disease control (CR + PR + SD) rates were 25.8% (23/89) and 55.1% (49/89), respectively, in this study. Because only 23 patients had objective responses, we only analyzed the association between disease control and clinical factors, and analyses of the association between objective response and clinical factors were not conducted. In the multivariate analyses using the original values, PPI non-use and PD-L1 TPS ≥ 50% were independent predictors of disease control (*P* = 0.0166 and *P* = 0.0277, respectively; **S4 Table**).

The IPTW-adjusted logistic analyses demonstrated that smoking, wild-type mutation status, PPI non-use, and PD-L1 TPS ≥ 50% were independent predictors of disease control (*P* = 0.0237, *P* = 0.0110, *P* = 0.0006, and *P* = 0.0243, respectively; **Table 4**). As presented in **Table 4**, PPI use had a stronger impact on disease control than did the PD-L1 expression status.

**Table 4:** Inverse probability of treatment weighting-adjusted univariate and multivariate analyses of the relationship between disease control (CR + PR + SD) and clinical factors.

Factors		Univariate analysis		Multivariate analysis	
		OR (95% CI)	*P* value	OR (95% CI)	*P* value
Age (years)	≥65/<65	0.62 (0.24–1.62)	0.3333		
Sex	Female/Male	0.40 (0.14–1.14)	0.0849		
ECOG PS	1–3/0	0.28 (0.11–0.69)	0.0057		
Smoking history	Never-smoker/Smoker	0.30 (0.10–0.87)	0.0270	0.21 (0.05–0.81)	0.0237
Mutation status (*EGFR* or *ALK*)	Others/Wild-type	0.24 (0.08–0.71)	0.0097	0.19 (0.05–0.68)	0.0110
Histology	Sq/Non-Sq	0.96 (0.32–2.87)	0.9388		
Probiotics	No/Yes	1.39 (0.40–4.87)	0.6041		
Proton pump inhibitor	Yes/No	0.19 (0.08–0.49)	0.0005	0.14 (0.05–0.43)	0.0006
PD-L1 tumor proportion score	<50%/≥50%	0.36 (0.14–0.89)	0.0278	0.28 (0.09–0.85)	0.0243

*ALK*, anaplastic lymphoma kinase; CI, confidence interval; CR, complete response; ECOG, Eastern Cooperative Oncology Group; *EGFR*, epidermal growth factor receptor; OR, odds ratio; PD-L1, programmed cell death-ligand 1; PR, partial response; PS, performance status; SD, stable disease; Sq, squamous cell carcinoma.

## Discussion

In this multicenter, retrospective study, we evaluated the impact of PPI use on clinical outcomes in patients with postoperative recurrent NSCLC receiving ICIs. The results of this study demonstrated that PPI use was significantly associated with poor clinical outcomes and that PPI use might reduce the efficacy of ICIs. These findings were similar to those of a previous report [9], but this is the first report demonstrating the effects of PPI use on the efficacy of ICIs in patients with postoperative recurrent NSCLC.

In the present study, the IPTW-adjusted Cox analyses showed that the ECOG PS, mutation status, PPI use, and PD-L1 expression status were independent prognostic factors for the PFS, whereas the ECOG PS, mutation status, probiotics use, and PPI use were independent prognostic factors for the OS. Furthermore, the IPTW-adjusted logistic analyses demonstrated that smoking, the wild-type mutation status, PPI non-use, and PD-L1 TPS ≥ 50% were independent predictors of disease control. The ECOG PS, smoking, mutation status, and PD-L1 were all previously reported to be significant predictive biomarkers of the efficacy of ICIs in NSCLC patients [18–25], which is similar to our results. PPI use had a stronger impact on the survival and disease control than the PD-L1 expression status, which is a strong predictor for selecting patients who might benefit from ICIs. Given these findings, PPIs should not be prescribed indiscriminately to patients with NSCLC who are expected to receive ICIs. The detailed mechanisms underlying the association between PPI use and ICI efficacy should be investigated in a future study.

Probiotics also influence the gastrointestinal microbiome, but the clinical impact of probiotic use on the outcomes of patients with NSCLC who receive ICIs is poorly understood. Therefore, we assessed the impact of probiotics in this study. Probiotic use was an independent favorable prognostic factor for the OS, but there was no significant association between probiotic use and the PFS or disease control. However, only 12 (12.6%) patients received probiotics in this study, and the results for probiotics only served as a reference in this study. In the future, we plan to analyze the impact of probiotic use on the efficacy of ICIs in a larger study cohort.

According to a previous report, PPI use alters both the intestinal flora and oral microbiome [26]. In the study, Mishiro et al. revealed the alterations of the microbiota in the oral carriage microbiome along with bacterial overgrowth (*Streptococcus*) along with decreases in the abundance of distinct bacterial species (*Neisseria* and *Veillonella*) [26]. For PPI use to be a useful factor in cancer immunotherapy, we should investigate the role of the oral microbiome in predicting the efficacy of ICIs.

There were several limitations associated with the current study. First, this was a retrospective study with a small sample size despite being a multicenter study. However, we conducted adjusted Kaplan–Meier survival analyses with the log-rank test, a Cox proportional hazards regression analysis, and a logistic regression analysis using IPTW to minimize the bias arising from the patients’ backgrounds in this study [16, 17]. The standardized mean differences of the whole model before and after weighting were 0.4257 and −0.0527, respectively, suggesting that the patients’ backgrounds were better balanced after IPTW. This study should be repeated in a larger cohort, and prospective studies may also be warranted. Second, we conducted analyses of the influence of PPIs on the clinical outcomes of postoperative recurrent NSCLC only in patients receiving ICIs in this study. We should conduct the same analyses including a control group comprising patients who were not treated with ICIs, as these results would be extremely informative. We also plan to repeat this analysis in a control group of patients who did not receive immunotherapy. Third, we did not examine how PPIs reduce the efficacy of ICIs in patients with recurrent NSCLC in this study, although we did mention the possible influence of PPIs on the gastrointestinal microbiome based on previous reports. We should clarify the detailed mechanisms underlying the influence of PPIs on the gastrointestinal microbiome in a future study. Fourth, approximately half of the patients who were prescribed PPIs were given the medicine without any apparent justification, a markedly high percentage. Most of them were given PPIs from another hospital, and we might be unable to obtain sufficient information about PPI use from these patients’ medical records. PPIs should not be prescribed indiscriminately to patients, regardless of ICI administration, as indiscriminate prescription of PPIs might cause various adverse events.

## Conclusions

In this study, PPI use in patients with postoperative recurrent NSCLC who received ICIs was significantly associated with poor clinical outcomes. PPIs are widely, and often excessively, used drugs. Some patients receiving PPIs had cancer pain, and they also received non-steroidal anti-inflammatory drugs; in addition, other patients had histories of reflux esophagitis or gastroduodenal ulcer. However, approximately half of the patients received PPI without obvious justification in this study. PPIs should not be prescribed indiscriminately to patients with postoperative recurrent NSCLC who are expected to receive ICIs. Furthermore, as indiscriminate prescription of PPIs might cause various adverse events, PPIs should not be prescribed indiscriminately to patients regardless of ICIs administration. The finding should be validated prospectively in a future study.

## Supporting information

S1 Table The breakdown ratios of the PPIs used for patients in this study. PPI, proton pump inhibitor.
(DOCX)

S2 Table Summary of the reasons for PPI among patients of this study (*N* = 37). PPI, proton pump inhibitor.
(DOCX)

S3 Table Univariate and multivariate analyses of PFS and OS using unadjusted values. PFS, progression-free survival; OS, overall survival.
(DOCX)

S4 Table Univariate and multivariate analyses of the relationship between disease control (CR + PR + SD) and clinical factors using unadjusted values. CR, complete response; PR, partial response; SD, stable disease.
(DOCX)

S1 Fig The Kaplan–Meier curves according to the use or non-use of proton pump inhibitors. (A) The progression-free survival and (B) the overall survival.
(TIF)
