# Supplementary material for: The clinical impact of concomitant medication use on the outcome of postoperative recurrent non-small-cell lung cancer in patients receiving immune checkpoint inhibitors
Source: PLoS One. 2022 Feb 7;17(2):e0263247. doi: 10.1371/journal.pone.0263247 (PMC8820612; doi:10.1371/journal.pone.0263247)
Supplement: S1 Table — PPI, proton pump inhibitor. (DOCX) [file pone.0263247.s001.docx]

**S1 Table.** The breakdown ratios of the PPIs used for patients in this study (*N* = 37)

| **PPIs** | **No. of patients** |
| --- | --- |
| Omeprazole | 0 (0.0%) |
|  |  |
| Lansoprazole | 12 (32.4%) |
|  |  |
| Rabeprazole | 7 (18.9%) |
|  |  |
| Esomeprazole | 16 (43.2%) |
|  |  |
| Vonoprazan fumarate | 2 (5.5%) |

PPI, proton pump inhibitor.
