# Supplementary material for: The clinical impact of concomitant medication use on the outcome of postoperative recurrent non-small-cell lung cancer in patients receiving immune checkpoint inhibitors
Source: PLoS One. 2022 Feb 7;17(2):e0263247. doi: 10.1371/journal.pone.0263247 (PMC8820612; doi:10.1371/journal.pone.0263247)
Supplement: S2 Table — PPI, proton pump inhibitor. (DOCX) [file pone.0263247.s002.docx]

**S2 Table.** Summary of the reasons for PPI among patients of this study (*N* = 37)

| **Reasons** | **No. of patients** |
| --- | --- |
| The patients received PPIs with NSAIDs for cancer pain. | 10 (27.0%) |
|  |  |
| The patients received PPIs with naproxen for tumor fever. | 1 (2.7%) |
|  |  |
| The patients received PPIs because of a history of gastroduodenal ulcer. | 5 (13.5%) |
|  |  |
| The patients received PPIs because of a history of reflux esophagitis. | 3 (8.1%) |
|  |  |
| The patients received PPIs without obvious justification. | 18 (48.7%) |

NSAID, non-steroidal anti-inflammatory drug; PPI, proton pump inhibitor.
