# Supplementary material for: The clinical impact of concomitant medication use on the outcome of postoperative recurrent non-small-cell lung cancer in patients receiving immune checkpoint inhibitors
Source: PLoS One. 2022 Feb 7;17(2):e0263247. doi: 10.1371/journal.pone.0263247 (PMC8820612; doi:10.1371/journal.pone.0263247)
Supplement: S3 Table — PFS, progression-free survival; OS, overall survival. (DOCX) [file pone.0263247.s003.docx]

**S3 Table.** Univariate and multivariate analyses of PFS and OS using unadjusted values

| **Factors** |  | **PFS** | | | | |  | **OS** | | | | |
| --- | --- | --- | --- | --- | --- | --- | --- | --- | --- | --- | --- | --- |
|  |  | **Univariate analysis** | |  | **Multivariate analysis** | |  | **Univariate analysis** | |  | **Multivariate analysis** | |
|  |  | **HR (95% CI)** | ***P* value** |  | **HR (95% CI)** | ***P* value** |  | **HR (95% CI)** | ***P* value** |  | **HR (95% CI)** | ***P* value** |
| Age (years) | ≥65/<65 | 1.11 (0.65–1.90) | 0.7037 |  |  |  |  | 1.20 (0.63–2.27) | 0.5781 |  |  |  |
|  |  |  |  |  |  |  |  |  |  |  |  |  |
|  |  |  |  |  |  |  |  |  |  |  |  |  |
| Sex | Female/Male | 1.40 (0.79–2.49) | 0.2483 |  |  |  |  | 1.12 (0.52–2.41) | 0.7672 |  |  |  |
|  |  |  |  |  |  |  |  |  |  |  |  |  |
|  |  |  |  |  |  |  |  |  |  |  |  |  |
| ECOG PS | 1–3/0 | 2.35 (1.46–3.78) | 0.0004 |  | 2.16 (1.23–3.81) | 0.0075 |  | 2.02 (1.14–3.57) | 0.0158 |  | 2.65 (1.44–4.88) | 0.0018 |
|  |  |  |  |  |  |  |  |  |  |  |  |  |
|  |  |  |  |  |  |  |  |  |  |  |  |  |
| Smoking history | Never-smoker/Smoker | 1.29 (0.72–2.32) | 0.3928 |  |  |  |  | 0.89 (0.40–1.99) | 0.7803 |  |  |  |
|  |  |  |  |  |  |  |  |  |  |  |  |  |
|  |  |  |  |  |  |  |  |  |  |  |  |  |
| Mutation status (*EGFR* or *ALK*) | Others/Wild-type | 1.79 (1.05–3.04) | 0.0315 |  | 2.20 (1.07–4.50) | 0.0316 |  | 1.65 (0.87–3.14) | 0.1275 |  |  |  |
|  |  |  |  |  |  |  |  |  |  |  |  |  |
|  |  |  |  |  |  |  |  |  |  |  |  |  |
| Histology | Sq/Non-Sq | 1.48 (0.85–2.56) | 0.1653 |  |  |  |  | 1.94 (1.06–3.55) | 0.0312 |  | 2.56 (1.34–4.87) | 0.0042 |
|  |  |  |  |  |  |  |  |  |  |  |  |  |
|  |  |  |  |  |  |  |  |  |  |  |  |  |
| Probiotics | No/Yes | 1.17 (0.56–2.45) | 0.6822 |  |  |  |  | 0.99 (0.44–2.21) | 0.9843 |  |  |  |
|  |  |  |  |  |  |  |  |  |  |  |  |  |
|  |  |  |  |  |  |  |  |  |  |  |  |  |
| Proton pump inhibitor | Yes/No | 1.79 (1.10–2.90) | 0.0181 |  | 3.28 (1.74–6.16) | 0.0002 |  | 1.74 (0.98–3.09) | 0.0587 |  |  |  |
|  |  |  |  |  |  |  |  |  |  |  |  |  |
|  |  |  |  |  |  |  |  |  |  |  |  |  |
| PD-L1 tumor proportion score | <50%/≥50% | 2.74 (1.48–5.09) | 0.0014 |  | 2.52 (1.33–4.77) | 0.0047 |  | 1.62 (0.77–3.42) | 0.2012 |  |  |  |
|  |  |  |  |  |  |  |  |  |  |  |  |  |

*ALK*, anaplastic lymphoma kinase; CI, confidence interval; ECOG, Eastern Cooperative Oncology Group; *EGFR*, epidermal growth factor receptor; HR, hazard ratio; OS, overall survival; PD-L1, programmed cell death-ligand 1; PFS, progression-free survival; PS, performance status; Sq, squamous cell carcinoma.
