# Supplementary material for: The clinical impact of concomitant medication use on the outcome of postoperative recurrent non-small-cell lung cancer in patients receiving immune checkpoint inhibitors
Source: PLoS One. 2022 Feb 7;17(2):e0263247. doi: 10.1371/journal.pone.0263247 (PMC8820612; doi:10.1371/journal.pone.0263247)
Supplement: S4 Table — CR, complete response; PR, partial response; SD, stable disease. (DOCX) [file pone.0263247.s004.docx]

**S4 Table.** Univariate and multivariate analyses of the relationship between disease control (CR + PR + SD) and clinical factors using unadjusted values

| **Factors** |  | **Univariate analysis** | |  | **Multivariate analysis** | |
| --- | --- | --- | --- | --- | --- | --- |
|  |  | **OR (95% CI)** | ***P* value** |  | **OR (95% CI)** | ***P* value** |
| Age (years) | ≥65/<65 | 0.66 (0.25–1.72) | 0.3925 |  |  |  |
|  |  |  |  |  |  |  |
|  |  |  |  |  |  |  |
| Sex | Female/Male | 0.78 (0.26–2.31) | 0.6539 |  |  |  |
|  |  |  |  |  |  |  |
|  |  |  |  |  |  |  |
| ECOG PS | 1–3/0 | 0.36 (0.15–0.85) | 0.0202 |  |  |  |
|  |  |  |  |  |  |  |
|  |  |  |  |  |  |  |
| Smoking history | Never-smoker/Smoker | 0.67 (0.22–2.03) | 0.4755 |  |  |  |
|  |  |  |  |  |  |  |
|  |  |  |  |  |  |  |
| Mutation status (*EGFR* or *ALK*) | Others/Wild-type | 0.31 (0.11–0.87) | 0.0257 |  |  |  |
|  |  |  |  |  |  |  |
|  |  |  |  |  |  |  |
| Histology | Sq/Non-Sq | 0.77 (0.28–2.09) | 0.6062 |  |  |  |
|  |  |  |  |  |  |  |
|  |  |  |  |  |  |  |
| Probiotics | No/Yes | 1.55 (0.44–5.52) | 0.4964 |  |  |  |
|  |  |  |  |  |  |  |
|  |  |  |  |  |  |  |
| Proton pump inhibitor | Yes/No | 0.54 (0.23–1.29) | 0.1642 |  | 0.28 (0.10–0.79) | 0.0166 |
|  |  |  |  |  |  |  |
|  |  |  |  |  |  |  |
| PD-L1 tumor proportion score | <50%/≥50% | 0.27 (0.09–0.80) | 0.0185 |  | 0.28 (0.09–0.87) | 0.0277 |
|  |  |  |  |  |  |  |

*ALK*, anaplastic lymphoma kinase; CI, confidence interval; CR, complete response; ECOG, Eastern Cooperative Oncology Group; *EGFR*, epidermal growth factor receptor; OR, odds ratio; PD-L1, programmed cell death-ligand 1; PR, partial response; PS, performance status; SD, stable disease; Sq, squamous cell carcinoma.
