# Supplementary figures and images for: The clinical impact of concomitant medication use on the outcome of postoperative recurrent non-small-cell lung cancer in patients receiving immune checkpoint inhibitors
Source: PLoS One. 2022 Feb 7;17(2):e0263247. doi: 10.1371/journal.pone.0263247 (PMC8820612; doi:10.1371/journal.pone.0263247)

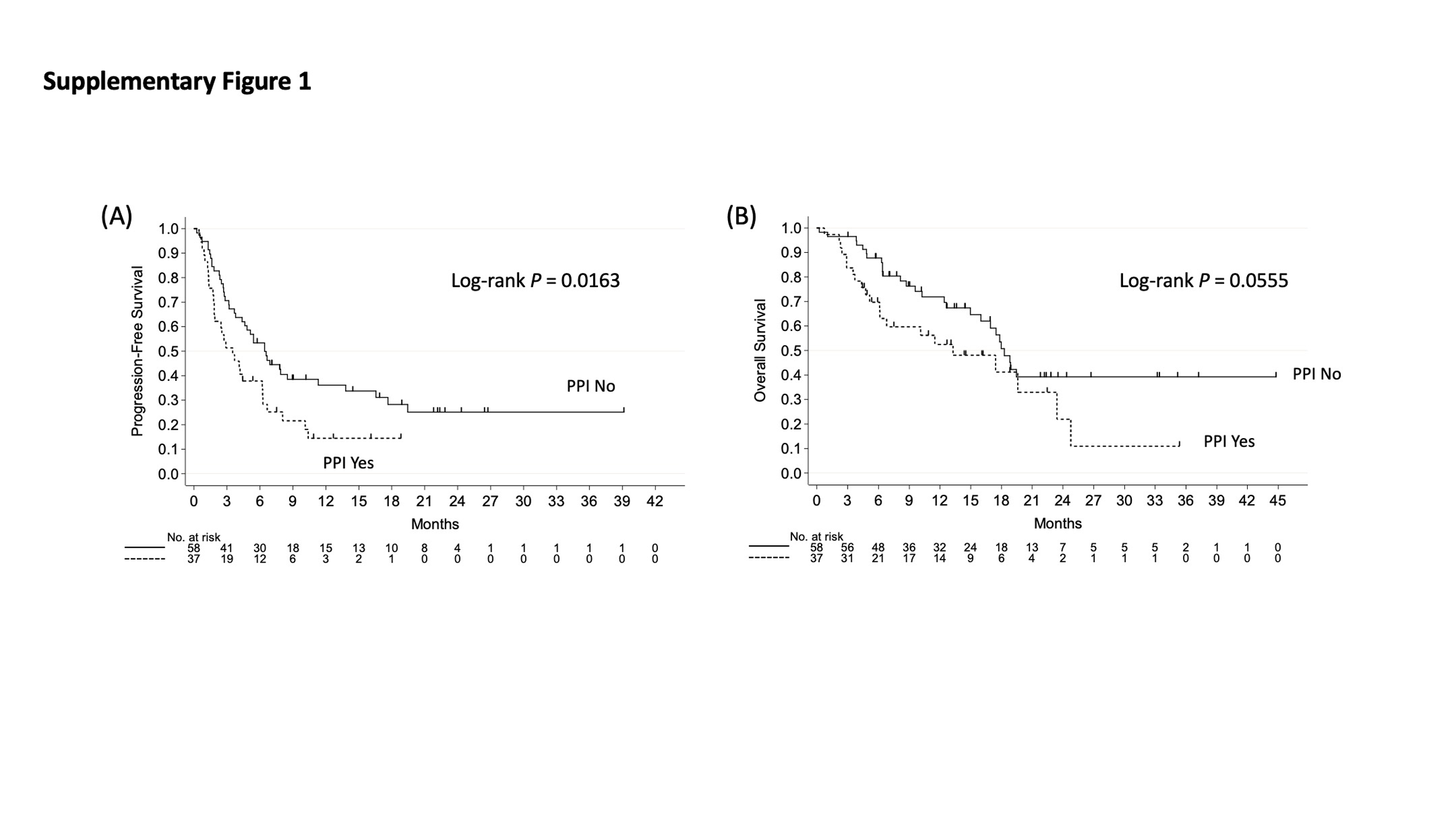

Supplement: S1 Fig — (A) The progression-free survival and (B) the overall survival. (TIF) [file pone.0263247.s005.tif]
